# Brain imaging data and summary data-based Mendelian randomization analysis reveal the impact of multiorgan aging on schizophrenia

**DOI:** 10.3389/fpsyt.2025.1730143

**Published:** 2026-02-02

**Authors:** Yan-Kun Han, Miao-Yan Liu, Ding-long Yang, Jia-Xin Xie, Xiao-Hui Wang, Dong-Bao Wang, Yun-Long Liang, Cui-Cui Wang, Long-Biao Cui, Yu-Jing Chen, Hai-Jun Zhang

**Affiliations:** 1 Department of Psychiatry, Xijing 986 Hospital, Fourth Military Medical University, Xi’an, China; 2 Shaanxi Provincial Key Laboratory of Clinic Genetics, Fourth Military Medical University, Xi’an, China; 3 Honghui Hospital, Xi’an Jiaotong University, Xi’an, China; 4 Unit 95871 of Air Force, Hengyang, China; 5 Department of Respiratory and Critical Care Medicine, The First Affiliated Hospital of Chongqing Medical University, Chongqing, China; 6 Department of Respiratory Medicine, The 988th Hospital of the Joint Logistic Support Force of the People’s Liberation Army of China, Zhengzhou, China; 7 Department of Radiology, The Second Affiliated Hospital of Xi’an Jiaotong University, Xi’an, China; 8 State Key Laboratory of Neurology and Oncology Drug Development, Xi’an, China

**Keywords:** aging, brain neuroimaging, brain-age, genome-wide association study, schizophrenia, transcriptome

## Abstract

**Aim:**

The adverse health outcomes of schizophrenia (SZ) are largely driven by the high prevalence of other non-neurological diseases. In addition to accelerated brain aging, patients with SZ also exhibit signs of systemic aging. However, the potential causal or biological mechanisms between multisystem aging and schizophrenia remain unknown.

**Methods:**

We obtained SZ-associated single-nucleotide polymorphism (SNP) sets, aging gene data, and tissue-specific cis-expression quantitative trait locus (cis-eQTL) data of the cerebral cortex and other tissues from a previous two-stage genome-wide association study (GWAS), GeneCards database, and Genotype-Tissue Expression (GTEx) project. We employed tissue-specific Mendelian randomization (MR) analysis to elucidate the tissue-specific expression patterns of aging-related genes, and used the summary data-based MR (SMR) approach to obtain tissue aging-related genes associated with the risk of SZ development. We identified the potential aging-related pathways through which these tissue-specific cis-eQTLs may affect SZ using enrichment analyses. Finally, we explored the relationship between the identified crucial aging-related genes and predicted age difference (PAD) of the brain in our clinical patients.

**Results:**

We found that the expression of tissue-specific aging genes, including synuclein alpha (*SNCA*), angiotensin I converting enzyme (*ACE*), BRCA1 DNA repair-associated (*BRCA1*), MutL homolog 1 (*MLH1*), vascular endothelial growth factor A (*VEGFA)*, microtubule-associated protein tau (*MAPT*), and age-related maculopathy susceptibility 2 (*ARMS2*), may affect SZ. The tissue-specific cis-eQTL may influence SZ through aging pathways. The brain PAD was significantly higher in the high-expression group of *BRCA1* than in the low-expression group.

**Conclusions:**

This study provides valuable clues to understand the link between SZ and multiorgan system aging and improves the current understanding of multiple tissue-specific aging-related genes with SZ.

## Introduction

Schizophrenia (SZ) is a highly heterogeneous disorder (1, 39). People with SZ show abnormalities in several organ systems in addition to the central nervous system (CNS) (2). To a large extent, the negative health outcomes for SZ are driven by the high rates of comorbid metabolic syndrome and related diseases (3). Previous studies have uncovered a shared genetic etiology among cardiovascular disease, frailty, and SZ, as well as altered oral microbiota and systemic immune dysfunction in patients with SZ (4–6). Whether SZ is a multisystem disorder or whether different mechanisms trigger its high rates of comorbidity but result from common risk factors still remains unknown.

Evidence suggests that aging plays an important role in SZ (7–10). Five case–control studies found that SZ is accompanied by accelerated biological aging by midlife (11). Previous studies have found that there is a common biological basis between SZ patients and normal elderly individuals with brain aging. In SZ and aging, astrocytes, glutamatergic, and GABAergic neurons show low synaptic neuron–astrocyte program expression, which is associated with cognitive flexibility and plasticity (12, 13). The cognitive impairment symptoms of SZ patients resemble those of the elderly, mainly involving decreased ability to process high-load information, episodic non-verbal memory impairment, slowed processing speed, and weakened motor coordination. These symptoms suggest that the pathological state of SZ patients is associated with accelerated brain aging (14–16). Compared with the general population, early SZ is associated not only with alterations in brain structure and function but also with multiple changes in the body (2). However, the association between the brain and body health, as well as the associated disease risk and physical multimorbidity across body systems, remains poorly characterized.

Mendelian randomization (MR) analysis is an emerging method that uses genetic variants as instrumental variables (IVs) to infer the causal effect of an exposure on an outcome (17). In order to be able to locate causality more precisely at the molecular level, we utilize the summary data-based Mendelian randomization (SMR), which could effectively integrate multisource data (18). Due to the specificity of IVs, the MR estimates are not commonly subject to confounding bias and reverse causation. MR has also been applied to detect putative causal effects of tissue-specific gene expression and a wide range of diseases using expression quantitative trait loci (eQTLs) as instruments (19, 20). Generally, comorbidity-related studies use SMR, which is essential for studying the causal relationships between different organ systems and diseases, as it helps to avoid confounding factors and establish more reliable causal links (21, 22).

The aim of this study was to investigate the causal effect of aging on SZ by using the MR method. To identify the potential target gene, the tissue-type-specific causal effects of aging on cognitive function were evaluated using cis-eQTL-based MR.

## Materials and methods

### Data acquisition

SZ-associated SNP sets were derived from a previous two-stage genome-wide association study (GWAS) (23). This is one of the largest available GWASs of SZ that report common variant associations at 287 distinct genomic loci, including up to 76,755 individuals with SZ and 243,649 control individuals. In the primary GWAS, they have analyzed up to 7,585,078 SNPs with MAF ≥1% in 175,799 individuals of whom 74.3% were European, 17.5% East Asian, 5.7% African-American, and 2.5% Latino. In the extended GWAS, they have meta-analyzed the primary GWAS results with summary statistics from deCODE Genetics (1,979 cases, 142,626 controls) for index SNPs with *P <*1 × 10^−5^ and identified 342 LD-independent significant SNPs located in 287 loci.

The tissue-specific cis-eQTL data of the brain cortex, brain hippocampus, brain hypothalamus, heart, liver, lung, kidney, pancreas, muscle, and adipose were obtained from the Genotype-Tissue Expression (GTEx) project (v8; https://gtexportal.org/home/). The GTEx Portal is a comprehensive public resource for researchers studying tissue- and cell-specific gene expression and regulation across individuals, development, and species, with data from three NIH projects. Ethical approval of all data was obtained in the original studies.

The aging genes were obtained from the GeneCards database (https://www.genecards.org/), which is a comprehensive and authoritative compendium of human gene information. We selected 50 genes to represent aging-related biology. These genes, which are often located at key nodes of aging molecular networks or broadly expressed in multiple aging-related tissues, correspond to the top 50 genes with the highest aging-related scores in the GeneCards database.

We recruited 43 patients with SZ from Xijing Hospital for brain MRI scanning to calculate brain age and collected peripheral whole blood samples from the patients to measure gene expression using RNA sequencing (RNA-seq) technology. The diagnosis of SZ was determined according to the Diagnostic and Statistical Manual of Mental Disorders, Fifth Edition (DSM-5) and confirmed by two experienced clinical psychiatrists after a comprehensive assessment of all available information. Detailed inclusion and exclusion criteria were previously documented. RNA-seq data derived from peripheral blood samples were utilized in this study, as previously described in detail. Specifically, 2.5 mL of whole blood was collected into PAXgene Blood RNA Tubes and immediately stored at −80°C. Subsequent RNA sequencing was performed on the Illumina NovaSeq 6000 platform. Raw sequencing data underwent quality control using Fastp (v.0.18.0; fastp: an ultra-fast all-in-one FASTQ preprocessor; https://github.com/OpenGene/fastp), with low-quality reads being excluded. The cleaned reads were then aligned to the human reference genome (hg19) using HISAT2.2.4 (HISAT: a fast spliced aligner with low memory requirements; https://daehwankimlab.github.io/hisat2/). Finally, the raw count data were normalized using DESeq2 (moderated estimation of fold change and dispersion for RNA-seq data with DESeq2; http://www.bioconductor.org/packages/release/bioc/html/DESeq2.html). The study protocol was reviewed and approved by the Xijing Hospital Institutional Ethics Committee and conformed to the ethical standards for medical research involving human subjects, as laid out in the 1964 Declaration of Helsinki and its later amendments. Participants provided written informed consent prior to taking part in the study.

### Brain age calculation

#### Imaging data acquisition and preprocessing

Patients and healthy controls underwent 3D high-resolution structural MRI scans, with raw images stored in DICOM format. Imaging data were acquired using a GE Discovery MR750 3.0T scanner and an 8-channel standard phased-array head coil (40).

#### Brain age model

Leveraging a 3D-convolutional neural network (3D-CNN) algorithm, this model accurately predicts brain age. Based on the classic VGGNet architecture, it is optimized into a simple fully convolutional neural network (SFCN) for efficient brain imaging data processing and analysis. Predicted age difference (PAD)=calculated age − chronological age. The specific establishment and training process of the brain age prediction model is detailed in the **Supplementary Material**.

### MR analysis

We first assessed different tissue-dependent effects of aging gene expression on SZ through tissue-specific MR analysis and estimated the putative causal effects of the expression of 50 aging genes in 10 tissues based on the GTEx database. MR analysis of the expression of aging genes in eight tissues on SZ was then conducted. FDR correction for SMR *P*-value was applied using the Benjamini–Hochberg method. Tissue-dependent effects of the expression of aging genes on SZ are significant when FDR__SMR_ <0.05 and *P*__HEIDI_ >0.05.

Then, we applied a tissue MR analysis to determine all tissue-specific eQTLs that have causal effects on SZ. The SMR selected tissue-specific cis-eQTL significantly associated with SZ with a genome-wide threshold of 5 × 10^−8^. All specific MR analysis details and codes are provided in the **Supplementary Material**.

### Enrichment analysis

Gene Ontology (GO) and Kyoto Encyclopedia of Genes and Genomes (KEGG) enrichment analyses were used to detect whether the tissue-specific cis-eQTLs through the aging pathways influence SZ. The “clusterProfiler” package in R software (https://guangchuangyu.github.io/software/clusterProfiler/) was utilized for the GO and KEGG enrichment analyses of cis-eQTL, with a selection criterion of *q*-value <0.05.

### Comparison of brain PAD between the high- and low-expression groups of aging genes

We divided the data of SZ patients into two groups, high- and low-expression, based on the top 25% and bottom 25% of tissue-specific senescence gene expression. Then, we analyzed their data on brain age to verify whether the screened genes significantly affect the brain PAD of SZ patients. A substantial brain age gap between the high- and low-expression groups points to this gene being a significant contributor to brain aging in schizophrenia patients.

### Statistical analyses

Data are presented as mean ± standard deviation (SD). Comparison between the two groups was performed using the Student’s *t*-test and the Wilcoxon test. All statistical analyses were conducted using SPSS software (version 30.0.0). A *P*-value of less than 0.05 was considered statistically significant.

## Results

### Tissue-dependent effects of aging gene expression on SZ

The MR analyses suggested putative causal effects of seven aging gene expressions in seven tissues on SZ. Among these were *ACE* in the lung, *VEGFA* in the pancreas, *MAPT* in the spleen, and *SNCA* in the heart’s left ventricle (FDR__SMR_ < 0.05, *P*__HEIDI_ > 0.05) (**Table 1**). Additionally, we detected several genes with suggestive associations, such as *MLH1* in the muscle and *BRCA1* in the liver.

**Table 1:** Tissue-dependent association for aging gene expression on SZ.

Tissue	ProbeID	ProbeChr	Gene	*P*_SMR	FDR_SMR	*P*_HEIDI
Lung	ENSG00000159640	17	*ACE*	2.28E−04	1.14E−03	6.05E−01
Pancreas	ENSG00000112715	6	*VEGFA*	8.78E−03	4.39E−02	4.95E−01
Spleen	ENSG00000186868	17	*MAPT*	2.27E−04	1.13E−03	1.77E−01
Heart	ENSG00000145335	4	*SNCA*	8.07E−03	4.04E−02	3.49E−02
Muscle	ENSG00000076242	3	*MLH1*	2.16E−02	1.08E−01	1.50E−01
Liver	ENSG00000012048	17	*BRCA1*	3.53E−02	7.06E−02	9.81E−01
Adipose	ENSG00000254636	10	*ARMS2*	7.26E−03	5.81E−02	8.38E−01

### The influence of tissue-specific aging eQTLs on SZ

**Figure 1:**
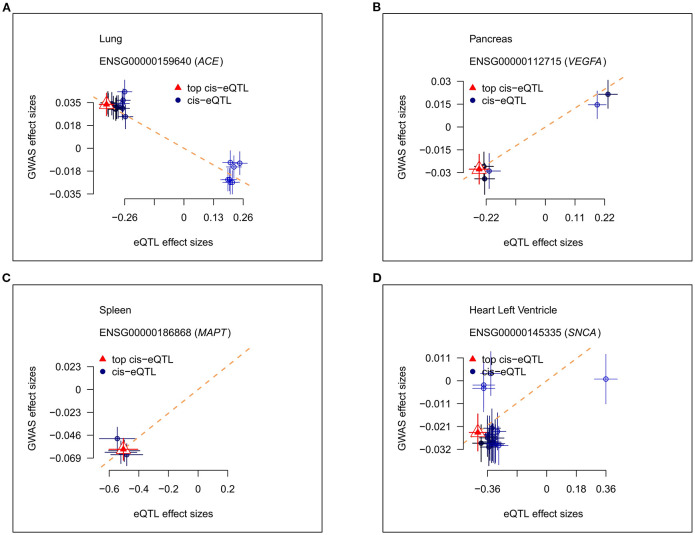
The relationship between the effect size of eQTLs and GWAS of tissue-specific genes. eQTL, expression quantitative trait locus; GWAS, genome-wide association study. **(A)** The relationship between the effect size of eQTLs and GWAS of tissue-specific genes in the lung. **(B)** The relationship between the effect size of eQTLs and GWAS of tissue-specific genes in the pancreas. **(C)** The relationship between the effect size of eQTLs and GWAS of tissue-specific genes in the spleen. **(D)** The relationship between the effect size of eQTLs and GWAS of tissue-specific genes in the left ventricle.

Among the seven tissues analyzed, we identified that the *ACE* gene in lung tissue (**Figure 1A**), the *VEGFA* gene in pancreatic tissue (**Figure 1B**), the *MAPT* gene in the spleen (**Figure 1C**), and the *SNCA* gene in the heart (**Figure 1D**) exhibited causal relationships with SZ.

### Enrichment analysis of tissue-specific eQTLs

**Figure 2:**
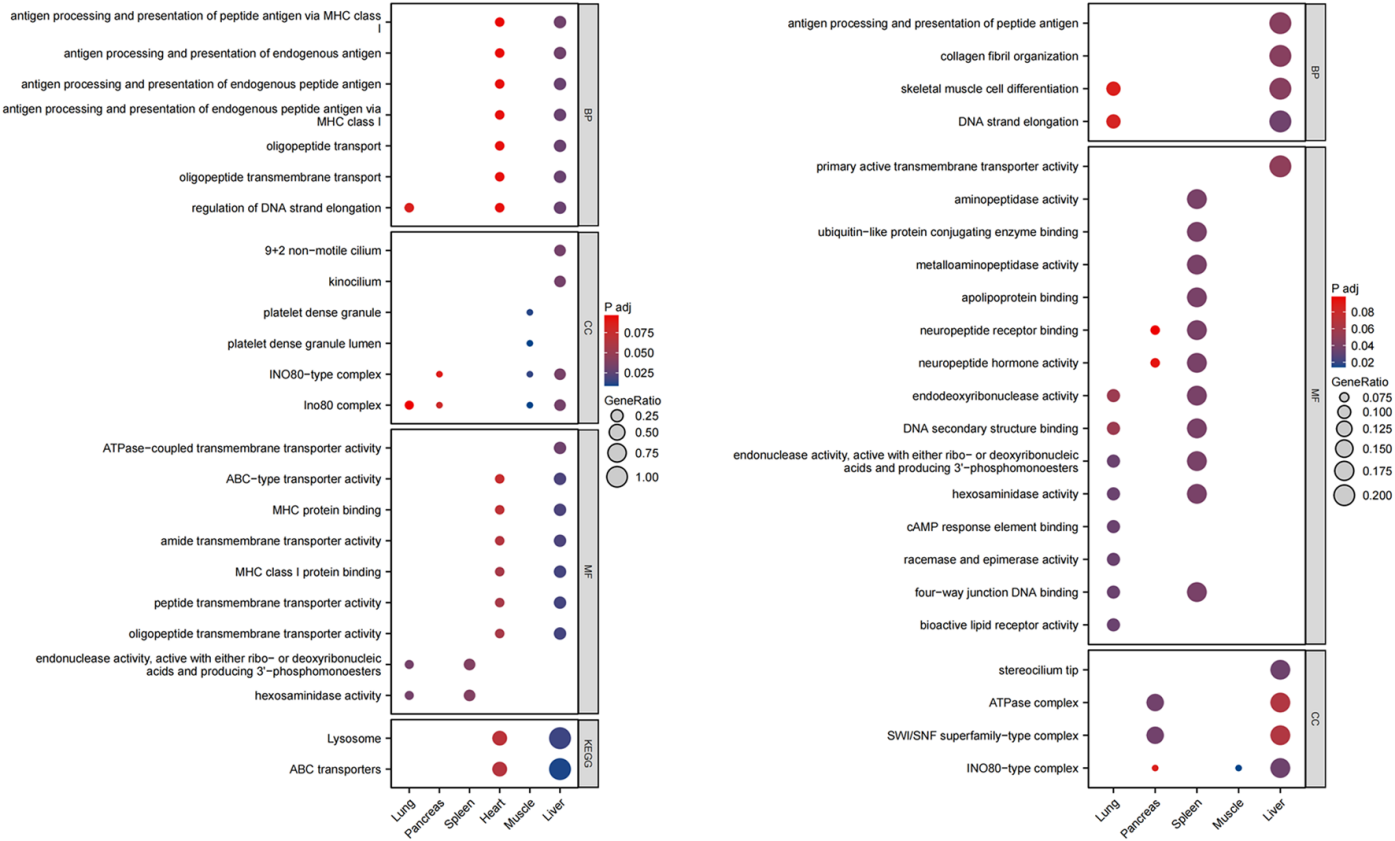
The GO and KEGG enrichment analyses of tissue-specific aging genes. GO, Gene Ontology. KEGG, Kyoto Encyclopedia of Genes and Genomes.

To further elucidate the connections between peripheral organs and SZ, we conducted enrichment analyses of tissue-specific genes associated with SZ (**Supplementary File 1**). As shown by the GO enrichment analysis, we found that organs like the liver, lungs, and heart may influence SZ through aging pathways. In the legend of **Figure 2**, GeneRatio was used to represent the proportion of tissue-specific genes annotated to different GO/KEGG pathways in an organ, while *P*_adj_ was used to represent the enrichment significance adjusted by the Benjamini–Hochberg procedure. For instance, liver-related pathways involve antigen processing and presentation (e.g., peptide antigen via MHC class I), DNA strand elongation, and oligopeptide transport. In the lungs, the cAMP-responsive element binding protein (CREB) pathway, the isomerase activity pathway, and the four-way junction DNA binding pathway are relevant. The pancreas-specific genes were associated with ATPase complexes and SWI/SNF superfamily type complex pathways. KEGG pathway analysis revealed that liver-specific genes associated with SZ were mainly enriched in lysosomes and ABC transporter proteins, which have essential roles in aging-related pathways. The details are shown in **Figure 2**.

### Validation of the effects of selected genes on brain PAD in SZ patients

**Figure 3:**
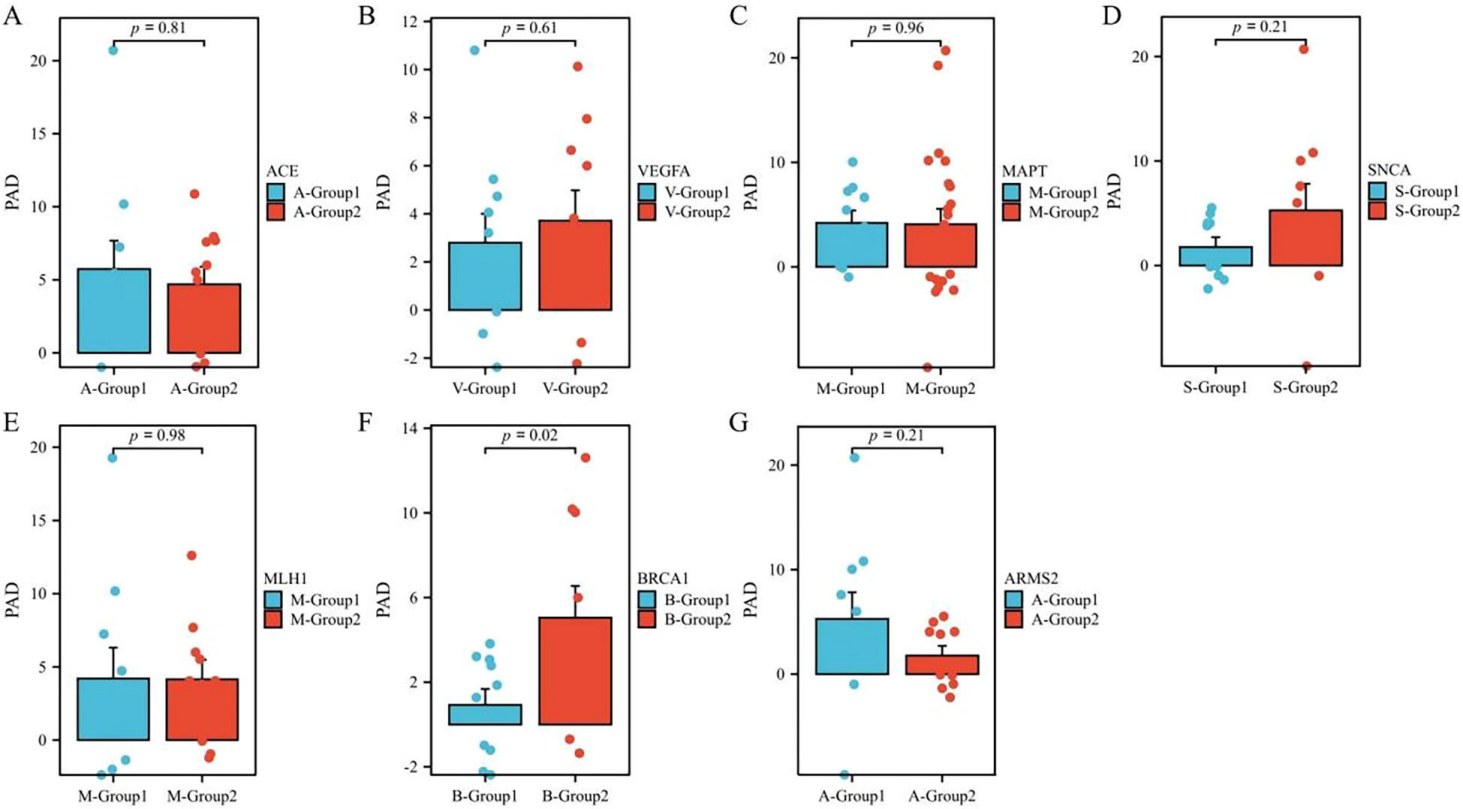
Analysis of the differences in brain PAD between the high- and low-expression groups of tissue-specific aging genes in patients with SZ. PAD, predicted age difference; SZ, schizophrenia. **(A)** Analysis of the differences in brain PAD between the high- and low-expression groups of *ACE* in patients with SZ. **(B)** Analysis of the differences in brain PAD between the high- and low-expression groups of *VEGFA* in patients with SZ. **(C)** Analysis of the differences in brain PAD between the high- and low-expression groups of *MAPT* in patients with SZ. **(D)** Analysis of the differences in brain PAD between the high- and low-expression groups of *SNCA* in patients with SZ. **(E)** Analysis of the differences in brain PAD between the high- and low-expression groups of *MLH1* in patients with SZ. **(F)** Analysis of the differences in brain PAD between the high- and low-expression groups of *BRCA1* in patients with SZ. **(G)** Analysis of the differences in brain PAD between the high- and low-expression groups of *ARMS2* in patients with SZ.

The brain PAD was significantly higher in the high-expression group of *BRCA1* than in the low-expression group (**Figures 3A–G**). Although statistically significant results were obtained only for the *BRCA1* gene, the *VEGFA* and *SNCA* still showed a favorable trend, probably due to the limitation of a small sample size.

## Discussion

Previous studies have mostly focused on the interplay between SZ and brain aging or the impact of a single organ or system on SZ. No research has yet examined how the body’s multiple organ systems may influence the brain via aging pathways to affect SZ. In this study, we used genetic tools and found that organ-specific aging genes in peripheral organs—*ACE* in the lung, *VEGFA* in the pancreas, *MAPT* in the spleen, *SNCA* in the heart’s left ventricle, *MLH1* in muscle, *BRCA1* in the liver, and *ARMS2* in adipose tissue—might influence SZ through aging-related pathways. Expression data of these genes in the whole blood and enrichment analysis results further confirmed this finding.

The *SNCA* gene encodes α-synuclein, which is involved in synaptic transmission and neurotransmitter release. α-Synuclein is highly expressed at the presynaptic terminal and is involved in a variety of cellular functions, including synaptic vesicle transport, membrane binding, and signal transduction, as well as affecting the homeostasis of the mitochondria and lysosomes (24). Joung et al. found that although the role of the *SNCA* gene is more pronounced in the brain, it also affects cardiac aging. The heart–brain axis is a bidirectional communication system that enables interaction between the heart and the brain through various pathways, including the autonomic nervous system and the immune system. Aging can lead to functional and structural changes in the heart, which in turn can affect brain function. A previous study found that cardiovascular diseases can result in reduced cerebral blood flow, potentially leading to cognitive decline symptoms observed in patients with SZ (25). Additionally, the *SNCA* gene may influence monocyte metabolism by participating in the regulation of the unfolded protein response and endoplasmic reticulum stress. Abnormal aggregation of α-synuclein may also activate monocytes and macrophages, prompting them to secrete pro-inflammatory cytokines and exacerbate systemic inflammation. These factors may all contribute to the development and progression of SZ. Our research also found that monocyte count genes are associated with cortical thickness (24, 26, 27).

The liver–brain axis and the muscle–brain axis are also bidirectional communication systems. Aging can lead to changes in liver function and structure, which may promote the development of liver and muscle diseases and affect brain function through the liver–brain and muscle–brain axes (28, 29). Although the primary functions of *BRCA1* and *MLH1* are related to DNA repair (30), they can still influence the function and structure of the liver and muscles, which in turn can affect brain function (31, 32). More importantly, in our clinical patients with SZ, the *BRCA1* gene expression in the blood is associated with brain PAD, which further suggests the potential role of the aging-related gene *BRCA1* in the liver–brain axis and SZ.

In lung–brain interaction, changes in *ACE* gene expression impact pulmonary blood flow and oxygenation, which in turn affect brain function. *ACE* inhibitors are widely used to treat hypertension and heart failure. Cao et al. found that the effects of ACE inhibitors on blood pressure can indirectly influence the lung–brain axis, thereby impacting brain function. Overexpression of *ACE* is associated with increased oxidative metabolism and heightened immune responses. This can affect the energy production and immune function of monocytes, leading to inflammation and subsequent brain changes, which is consistent with our study (27, 33).

*ARMS*2 is a gene linked to age-related macular degeneration. It encodes a secreted protein functioning in the extracellular matrix, likely maintaining matrix homeostasis through interactions with various matrix proteins (34). Adipose tissue undergoes significant changes during aging, including adipocyte volume reduction, decreased lipid storage capacity, and alterations in adipokine secretion levels (35). Adipose tissue may upregulate *ARMS2* to regulate and maintain its structure and function. *ARMS2* may interact with matrix proteins to influence monocyte migration, phagocytosis, and immune responses. Its mitochondria-associated functions could indirectly affect monocyte energy metabolism and activity (36), potentially causing chronic inflammation that impacts brain function (27).

The *VEGFA* gene is crucial for angiogenesis, inflammation, and oxidative stress. As pancreatic tissue ages, vascular regression and reduced blood supply occur. By promoting angiogenesis, *VEGFA* may improve pancreatic blood supply and slow its aging. *VEGFA* might also alter chromatin accessibility via the SWI/SNF complex, regulating genes linked to neuronal survival, differentiation, and synaptic plasticity. Moreover, *VEGFA* overexpression may activate pathways that influence monocyte and macrophage function, potentially leading to a pro-inflammatory microenvironment (27, 37, 38).

However, one limitation of the present study is the relatively modest sample size (*n*=43) of the clinical cohort employed for the validation analyses examining the relationship between peripheral aging-related gene expression and brain PAD in schizophrenia patients. This limited sample size may have constrained statistical power, and future investigations incorporating multicenter collaborations and expanded sample sizes are essential to validate our preliminary observations.

In conclusion, our study offers novel insights into the relationships between SZ and the aging-related genes *SNCA*, *ACE*, *BRCA1*, *MLH1*, *VEGFA*, *MAPT*, and *ARMS2* in multiple organs. Notably, the *BRCA1* gene may be associated with accelerated brain aging in individuals with SZ. Our findings provide valuable clues for understanding the link between peripheral organ aging and SZ.

## Data Availability

The datasets presented in this study can be found in online repositories. The names of the repository/repositories and accession number(s) can be found in the article/**Supplementary Material**.
