# Supplementary material for: Brain imaging data and summary data-based Mendelian randomization analysis reveal the impact of multiorgan aging on schizophrenia
Source: Front Psychiatry. 2026 Feb 2;16:1730143. doi: 10.3389/fpsyt.2025.1730143 (PMC12907309; doi:10.3389/fpsyt.2025.1730143)
Supplement: Supplementary file 1 [file DataSheet1.docx]

Supplementary Material

Content

Architecture and training procedure of the brain age prediction model 2

**Specific details of MR analysis 4**

**Core code for the Mendelian randomization process 6**

Architecture and training procedure of the brain age prediction model

*Data preprocessing*

During sMRI data acquisition, in order to eliminate the effects of differences in acquisition sites, imaging equipment, and acquisition parameters on the images, it is necessary to preprocess the data of each subject in order to eliminate noise and to calibrate the differences between different imaging time points.The preprocessing of the sMRI data of the subjects in this study was accomplished using the FSL software.The preprocessing process includes alignment, removal of skull and non-brain tissue, and bias correction.
(1) Alignment: Since the image acquisition time of each layer of sMRI data is different during the scanning process, interlayer displacement, deformation, and signal intensity differences may occur. Therefore, alignment operations are required to improve the comparability and consistency of the data among different modalities or images from different sources, which include interlayer correction, temporal correction, cephalic motion correction, and spatial normalization.In this study, we used FSL software to normalize the brain sMRI images of all subjects to the MNI152 (MNI152_T1_1mm) template developed by the Montreal Neurological Institute (MNI).

(2) Skull removal: sMRI raw data contains a large number of non-brain tissue structures, which requires removing the skull and other non-brain structures and retaining only the brain tissue. In this study, the BET algorithm in FSL software was used to segment and remove non-brain and brain tissues based on the gray scale difference and texture characteristics between skull and brain tissues, in which the frac parameter was set to the default value of 0.5.

(3) Bias correction: There are geometrical aberrations, tissue signal differences, and noise interferences in sMRI data, and it is necessary to carry out a bias correction on the MRI data, which is aimed at correcting image. The purpose of bias correction is to correct the bias and distortion in the image to improve the accuracy and reliability of the subsequent analysis.Bias correction usually involves correcting geometric distortion, tissue signal differences, and noise interference in the images. Geometric correction of images using nonlinear transformation methods to correct geometric distortions due to head pose, scanning equipment, etc. Correction methods based on grayscale or tissue signal differences are used to eliminate signal strength differences between different tissues and make the image more uniform and consistent.

*Brain Age Prediction Model*

We use 3D-CNN algorithm to predict brain age based on high-resolution T1 structural images. The model is based on VGGNet and adopts a fully convolutional structure, which combines the advantages of VGGNet and fully convolutional networks, and mainly consists of three layers: a feature extraction layer (consisting of five identical blocks, which can improve the feature extraction and generalization ability of the model, and each block contains a 3D convolutional layer, a batch normalization layer, a maximum pooling layer and a ReLU activation function)) Nonlinear enhancement layer (contains a 3D convolutional layer with a convolutional kernel size of 1×1×1, a batch normalization layer, and a ReLU activation function) Brain age prediction layer (contains an average pooling layer, a dropout layer, a 3D convolutional layer, and a softmax output layer).

*Training set*

Since the brain sMRI data are important for the construction of the model for the prediction of the brain age, a large number of multicenter samples are used for the modeling of the brain age prediction. The training set of this study consists of brain imaging datasets from several centers, including the publicly available Information eXtraction from Images (IXI) database, the Cambridge Centre for Ageing and Neuroscience (Cam-CAN) database, the Southwest University Adult Lifespan Dataset (SALD), the Open Access Series of Imaging Studies (OASIS), and the Schizophrenia Imaging Lab (SIL) at the Air Force Medical University.

Specific details of MR analysis

We first extracted the senescence genes of each organ and then did SMR analysis with the GWAS data of schizophrenia patients to obtain the tissue senescence-related genes associated with the risk of developing schizophrenia (SZ).

*Instrumental Variable Selection: Definition of cis-eQTLs*

For each of the 50 pre-selected aging-related genes, tissue-specific cis-eQTLs were extracted from the GTEx v8 database across 10 somatic tissues (brain cortex, hippocampus, hypothalamus, heart, liver, lung, kidney, pancreas, muscle, and adipose). cis-eQTLs were defined using the following stringent criteria:

(1) Genomic Window: SNPs located within ±1 Mb of the gene's transcription start site (TSS) were included.

(2) Statistical Significance: Only eQTLs with q-value < 0.05 (Benjamini–Hochberg corrected within each tissue) were retained to ensure tissue-specific regulatory effects.

(3) For each gene-tissue pair, the most significant SNP surviving this pruning (i.e., the "top variant") was selected as the primary IV.

*Instrument Strength Assessment*

The strength of each IV was quantified using the F-statistic, calculated as F = β²ₑQTL / SE²ₑQTL, where βₑQTL and SEₑQTL denote the effect size and standard error of the eQTL association. Only IVs with F-statistic > 10 (range: 23.4–1,847.6) were included in downstream analyses, thereby minimizing bias from weak instruments.

*Primary Analysis: Single-SNP Summary-data-based MR (SMR)*

Causal estimation was performed using the SMR v1.3.1 software (https://yanglab.westlake.edu.cn/software/smr/). For each gene-tissue combination, we integrated:

Exposure: eQTL summary statistics from GTEx v8.

Outcome: SZ GWAS summary statistics from Trubetskoy et al. (2022) Nature (up to 76,755 cases and 243,649 controls).

LD Reference: Pairwise LD matrices derived from the 1000 Genomes Project Phase 3 EUR super-population $n=503$, consistent with the predominant ancestry (74.3% European) of the GWAS cohort.

To exclude horizontal pleiotropy, we applied the HEIDI test (default parameters: maximum LD r² = 0.9, window size = ±5 Mb). Associations were considered significant only if P_SMR < 0.05 and P_HEIDI > 0.05. Cross-gene FDR correction within each tissue was applied using the Benjamini–Hochberg method, with a significance threshold of FDR < 0.05.

Core code for the Mendelian randomization process

We used R when we organized the GWAS data in the front of the study, and we also used R when we extracted the related genes in the QTL dataset for smr analysis and graphing in the back. The main code is as follows:

*Extraction of related genes in the QTL dataset*

./smr-1.3.1 --beqtl-summary ./血液/eQTL原始/eQTL --query 1 --genes gene.list --out multiple --make-besd

*SMR analysis (GWAS-QTL)*

./smr-1.3.1 -.bfile ./g1000 eur/q1000 eur -qwas-summany ./sc/sc.txt -begt-summary ./血液/eQTL/multiple --out mysmr --thread-num 10
